# The atomic portrait of SARS‐CoV‐2 as captured by cryo‐electron microscopy

**DOI:** 10.1111/jcmm.17103

**Published:** 2021-12-14

**Authors:** Tudor Emanuel Fertig, Leona Chitoiu, George Terinte‐Balcan, Victor‐Eduard Peteu, Daciana Marta, Mihaela Gherghiceanu

**Affiliations:** ^1^ Ultrastructural Pathology and Bioimaging Lab Victor Babeș National Institute of Pathology Bucharest Romania; ^2^ Carol Davila University of Medicine and Pharmacy Bucharest Romania

**Keywords:** COVID‐19, cryo‐electron microscopy, cryo‐electron tomography, SARS‐CoV‐2, single‐particle analysis, structural biology

## Abstract

Transmission electron microscopy has historically been indispensable for virology research, as it offers unique insight into virus function. In the past decade, as cryo‐electron microscopy (cryo‐EM) has matured and become more accessible, we have been able to peer into the structure of viruses at the atomic level and understand how they interact with the host cell, with drugs or with antibodies. Perhaps, there was no time in recent history where cryo‐EM was more needed, as SARS‐CoV‐2 has spread around the globe, causing millions of deaths and almost unquantifiable economic devastation. In this concise review, we aim to mark the most important contributions of cryo‐EM to understanding the structure and function of SARS‐CoV‐2 proteins, from surface spikes to the virus core and from virus‐receptor interactions to antibody binding.

## 1 INTRODUCTION

A novel severe acute respiratory syndrome coronavirus (SARS‐CoV‐2) was identified in late 2019 as the infectious agent responsible for coronavirus disease 2019 (COVID‐19),^1^ which most frequently not only presents with respiratory symptoms of varying severity, but can also affect other organs. Due to the relatively high basic reproductive number of the virus,^2^ what initially started as an outbreak in Wuhan, Hubei province, China, rapidly became a pandemic, spreading to all countries, infecting over 250 million people and killing over 5 million as of November 2021.^3^ Understanding how SARS‐CoV‐2 functions immediately became a priority for a large part of the global scientific community.

Historically, transmission electron microscopy (TEM) has proven indispensable for diagnosing disease caused by known and novel viruses and for understanding the mechanisms underlying infection.^4^, ^5^, ^6^ In the past decades, as cryo‐electron microscopy (cryo‐EM) developed and became more accessible, obtaining near‐atomic resolution 3D structures of viral components or even whole viruses became a standard in the field of molecular virology.^7^, ^8^, ^9^

In this review, we condense almost two years of cryo‐EM research on the structure and function of SARS‐CoV‐2 proteins in a concise and accessible way, even for those not trained in structural biology. At first glance, it may seem that mostly selecting studies which employ cryo‐EM would reduce the ability of this review to tell a coherent story. To the contrary, cryo‐EM is currently almost indispensable to understanding viral mechanics, from how SARS‐CoV‐2 infects cells to how new mutations allow it to escape neutralizing antibodies.

## 2 THE STRUCTURAL PROTEINS OF SARS‐COV‐2

### 2.1 N‐M‐E‐S is the A‐B‐C of SARS‐CoV‐2 structure

SARS‐CoV‐2 is a large, enveloped, positive‐sense and single‐stranded RNA virus in the family *Coronaviridae*, which includes SARS‐CoV‐1 and MERS‐CoV, but also the less threatening ‘common‐cold’ human coronaviruses, OC43, HKU1, NL‐63 and 229E. As is the case with other members of the family, the genome of SARS‐CoV‐2 is packaged with the help of nucleocapsid (N) proteins and contained within a lipid bilayer, which incorporates a transmembrane envelope (E) protein and an associated membrane (M) protein. The E and M proteins are both mainly involved in the assembly of virions within the endoplasmic reticulum Golgi intermediate compartment (ERGIC) of the host cell and induce the required membrane curvature of budding virions.^10^

Anchored in the lipid bilayer are the spike (S) glycoproteins (hereafter referred to as S‐proteins), inarguably the most scrutinized structural component of SARS‐CoV‐2. The S‐protein trimer, formed by the association of three identical protomers, strongly binds to the human angiotensin‐converting enzyme 2 (ACE2) receptor, found on the surface of many cell types, notably alveolar cells in the lung, enterocytes in the small intestine and endothelial cells.^11^ An average of 24 S‐protein ectodomains unevenly decorate each virion,^12^ giving SARS‐CoV‐2 the characteristic ‘crown‐like’ appearance. Each S‐protein is roughly 20 nm in length and club‐shaped, with a relatively wide head region connected to the viral membrane through a thin stalk.^13^

Functionally, each protomer of the S‐protein comprises two regions: (1) S1, which contains an N‐terminal domain (NTD) and the receptor‐binding domain (RBD), and (2) the C‐terminal S2, which harbours a fusion protein (FP) and is responsible for fusion with the target‐cell membrane. SARS‐CoV‐2 cell entry is made possible by conformational changes in the S‐protein, which switches from a prefusion to a postfusion state following cleavage by host‐cell proteases and shedding of S1 (as detailed further).

### 2.2 S‐protein structure resolved in record‐breaking time

SARS‐CoV‐2 is pleomorphic, with virions varying in diameter from 80 to 140 nm.^12^, ^14^ The size and even shape variations of individual virions make it virtually impossible to reconstruct the entire virus using typical cryo‐EM image processing. Instead, smaller regions of whole virions can be aligned and averaged from cryo‐electron tomography (cryo‐ET) data to generate high‐resolution 3D reconstructions. More often, pleomorphic viruses have to be broken down to their individual soluble and symmetrical proteic components, which can then be imaged by cryo‐EM and digitally reconstructed at near‐atomic resolution using *single*‐*particle analysis* (SPA) workflows. This adds a layer of complexity, as it requires expression and purification of the recombinant viral protein of interest, prior to cryo‐EM data acquisition.

Nevertheless, at the beginning of March 2020, just as the World Health Organization was declaring COVID‐19 a pandemic,^15^ two seminal papers were published solving the 3D structure of the isolated, recombinant S‐protein trimer by cryo‐EM SPA. One was from the laboratory of Jason McLellan at the University of Texas^16^ and another from the David Veesler's laboratory at the University of Washington.^17^ The authors resolved the structure of a proline‐stabilized prefusion conformation at 3.5 Å and 2.8 Å resolutions, respectively, revealing structural homology to the known SARS‐CoV‐1 S‐protein, including intrinsic flexibility of the RBD.^16^, ^17^ The structures suggested similar mechanisms of activation and subsequent membrane fusion and cell entry for the two SARS viruses^16^ and potential cross‐reactivity with antibodies for other coronaviruses.^17^

They also highlighted a key feature of S‐protein immunogenicity: the presence of a glycan shield, resulting from 66 potential N‐linked glycosylation sites per trimer (or 22 per protomer).^16^, ^17^ This not only serves to hide some S‐protein epitopes from detection by the host immune system, but also contributes to folding and protease interactions.^17^ Conversely, the lesser glycan coverage of the RBD helps explain why the overwhelming majority of neutralizing antibodies are generated against this region.^18^

**FIGURE 1:**
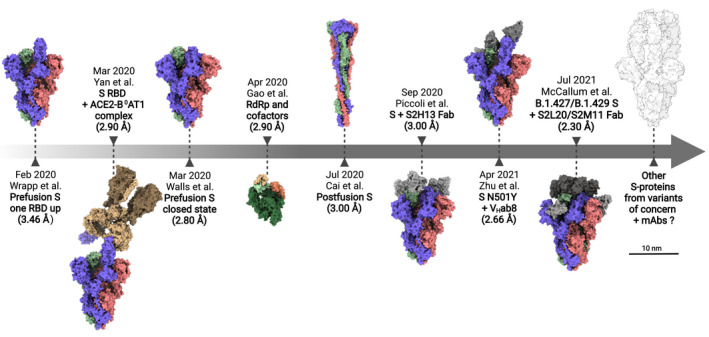
Brief timeline of cryo‐EM milestones in the study of SARS‐CoV‐2. Diagrams are shown to scale. PDB structures from left to right: 6VSB, 6M17, 6VXX, 6M71, 6XRA, 7JV6, 7MJH and 7N8H. Each S‐protein protomer is shown in a different colour (purple, red and green), whereas antibodies bound to the S‐protein are shown in shades of grey

Interestingly, just two states of the S‐protein trimers are described between the two studies (Figure 1): one in which all protomers are in a closed or partially closed conformation (the ‘down’ position of the RBD, PDB 6VXX), thereby hiding the receptor‐binding motifs (RBMs) and another with one of the three RBDs open or exposed (the ‘up’ position of the RBD, PDB 6VSB and 6VYB).^16^, ^17^ In fact, soluble constructs with more than one ‘up RBD’ could only be obtained through the introduction of multiple mutations.^19^ These preferred conformations may allow an optimal compromise between a biologically active, fusion‐prone state and a closed state favouring immune evasion. Indeed, another cryo‐EM study later revealed that at endosomal and lysosomal pH, an aspartic acid‐rich region located between adjacent protomers can act like a molecular switch, that leads to retraction of RBDs. This fully closed conformation resists antibody binding and may even shed bound antibodies during cell entry via endosomes.^20^

Overall, these early structures represented an essential first step in understanding the mechanisms of ACE2 binding by SARS‐CoV‐2 and, importantly, paved the way for the development of potential therapeutics or vaccines targeting the RBD. Remarkably, the two studies were submitted for publication in February, just a month after the first version of the SARS‐CoV‐2 viral genome was uploaded to GenBank (MN908947).^21^ This was a record‐breaking achievement, showcasing not only the evolution of molecular biology methods, but also the power of the currently available cryo‐EM technology and the rapid development of highly standardized SPA workflows. By comparison, although a 16 Å structure for the entire SARS‐CoV‐1 S‐protein was published in 2006^22^ and smaller regions were resolved before that, complete high‐resolution structures of its different conformations were only obtained in late 2016^23^ and 2017,^24^ more than a decade after virus emergence.

### 2.3 The S‐protein specifically targets the ACE2 receptor

Almost immediately after the structure of the S‐protein was solved, another cryo‐EM study revealed the interaction between the SARS‐CoV‐2 RBD and a B^0^AT1‐stabilized ACE2 human receptor, suggesting that ACE2 is a homodimer which can simultaneously bind two S‐protein trimers (Figure 1, PDB 6M17).^25^ Conversely, each S‐protein can bind up to three ACE2 ectodomains.^20^, ^26^ It was later shown that binding to ACE2 is facilitated by the intrinsic flexibility of the S‐protein ectodomain, which has three ‘hinge‐regions’ in the stalk, allowing the head to tilt up to 90°.^13^ These molecular joints would therefore compensate for virion positioning and variations of cell membrane topography.^13^ The RBD itself has also been shown to tilt against the axis of the S‐protein during ACE2‐coupling.^26^

**FIGURE 2:**
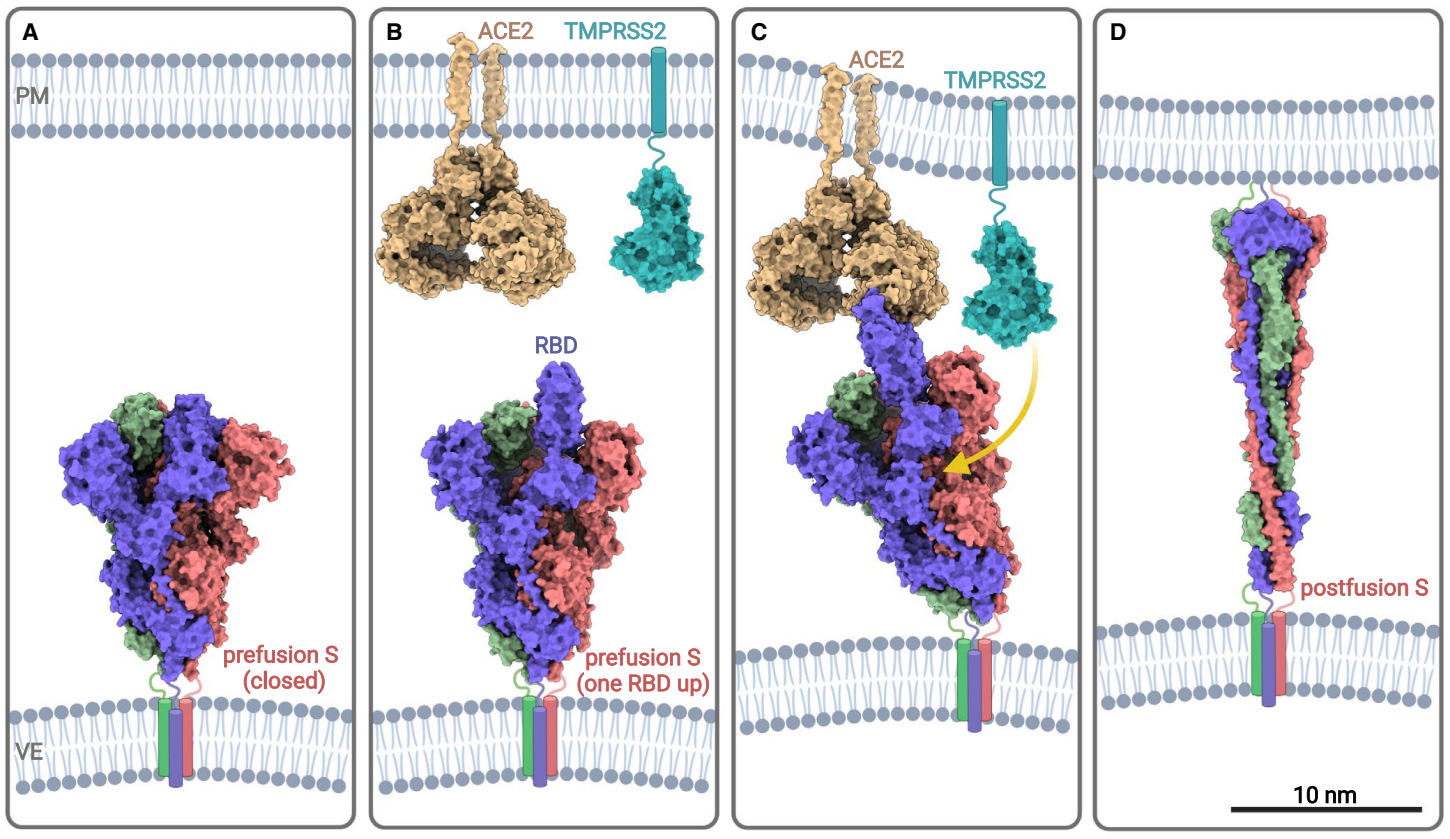
Proposed conformational changes in the SARS‐CoV‐2 S‐protein leading to membrane fusion (adapted from^33^, ^34^). (A) Most S‐proteins on virions are in a ‘closed’ or prefusion state, shielding the RBDs from immune surveillance; (B) Some S‐proteins are pre‐activated, with one exposed RBD for ACE2‐coupling—possibly a result of cleavage by furin at the S1/S2 boundary during biosynthesis; (C) Binding to ACE2 changes the molecular architecture of the S‐protein to a more open state, with progressive engagement of the remaining two RBDs. Plasma membrane proteases, such as furins or TMPRSS2 (PDB 7MEQ), cleave the now‐exposed S2’ region allowing dissociation of the S1 subunits; (D) The elongated and rigid postfusion S‐protein closes the gap between the two membranes, leading to fusion and cell entry (PDB 6XRA). Diagrams are shown to scale (S—*spike protein*, RBD —*receptor*‐*binding domain*, PM—*plasma membrane*, VE—*viral envelope*, ACE2—*angiotensin*‐*converting enzyme 2*, TMPRSS2 ‐ *transmembrane serine protease 2*)

Cryo‐EM contributed to our understanding that the SARS‐CoV‐2 RBD binds the human ACE2 receptor with more affinity, but in a structurally similar way compared with some bat^27^ and even cat ACE2 orthologs,^28^ reinforcing bats as the likely primordial host for the virus, while also suggesting a very broad host range. However, the interactions between the S‐protein and the ACE2 receptor are complex and binding assays using isolated RBDs do not always tell the whole story. For example, the isolated RBD of SARS‐CoV‐2 binds human ACE2 with higher affinity than the RBD of SARS‐CoV‐1; however, strength of binding is essentially reversed when using the entire S‐proteins.^29^ This is likely due to SARS‐CoV‐2 RBDs being more hidden by the ‘closed state’ of the trimers, thereby making them less accessible for both ACE2 binding, as well as the host immune system (Figure 2A).^19^, ^29^ SARS‐CoV‐2 overcomes this apparent affinity disadvantage by undergoing successive conformational changes in the S‐protein, starting from its biosynthesis to the moment of contact with the ACE2 receptor. These changes, revealed by cryo‐EM, gradually and irreversibly shift the molecular architecture of the S‐protein from the prefusion to the postfusion state, to allow cell entry.

### 2.4 The S‐protein requires proteolytic cleavage for activation

An initial conformational change occurs when some S‐proteins are pre‐activated by furins localized within the secretory pathway of the cell of origin. During viral assembly, these furins cut at a PRRAR furin‐cleavage site (FCS) located on the S1/S2 boundary, a process suggested to promote disordering and then exposure of the RBD for ACE2 binding (Figure 2B).^30^ The FCS is notably absent in SARS‐CoV‐1, which is cleaved at S1/S2 by trypsin‐like proteases at the cell surface (*ie* transmembrane serine protease 2, TMPRSS2).^31^

The second, critical conformational change is triggered by the coupling of the S‐protein to the ACE2 receptor. The fact that ACE2 is indispensable for S‐protein activation is supported by cryo‐ET observations showing that virions found in contact with cells expressing low levels of ACE2 showed a predominance of trimers in a prefusion state, whereas virions in proximity of cells expressing higher levels of ACE2 had more postfusion spikes.^32^

Indeed, when S‐proteins with one exposed RBD bind to ACE2, a structural shift of the trimer to a fully open state is initiated, which sees the successive engagement of the remaining two RBDs (Figure 2C)^26^, ^33^ Initially, conformational changes in the S2 region itself may be subtle^20^; however, as more RBDs bind, the trimer structure loosens and the S1 domains are pushed away from each other, thus activating the FP and exposing the S2 core.^26^, ^33^ This process also serves to unmask the second, synergistic cleavage site in the S2’ region,^34^ vulnerable to the activity of furins or TMPRSS2 that localize at the surface of the plasma membrane (Figure 2C). Proteolytic cleavage at S2’ leads to final refolding of the S2 domain, exposing the FP and allowing membrane fusion.^34^ It remains unclear how many ACE2 receptors need to be bound to each trimer to induce the conformational changes required for efficient fusion.^26^

Notably, there is less coverage by the S‐protein glycan shield in close proximity to both the S1/S2 and the S2’ cleavage sites, to allow unhindered proteolysis.^33^

Later in 2020, the structure of the postfusion conformation of the S‐protein was solved by three studies, one at 3 Å resolution using single‐particle cryo‐EM on expressed and purified S‐proteins (Figure 1, PDB 6XRA),^34^ and another two using cryo‐ET on intact virions, at 11 Å and 15.3 Å, respectively.^14^, ^35^ The studies converge on a ‘nail‐like’ structure for the remaining S2 trimer,^14^ defined by a central, elongated and rigid three‐stranded coiled‐coil, which forms following proteolytic cleavage at the S2’ site and dissociation of S1 (Figure 2D).^34^ Interestingly, a comparison of these cryo‐ET studies suggests that inactivation of virions using β‐propiolactone may shift a majority of S‐proteins to a postfusion conformation,^14^ a phenomenon not observed for formaldehyde inactivation.^12^, ^13^, ^35^

Despite this already highly efficient cascade of events, SARS‐CoV‐2 infection presents redundancies, which make development of targeted therapies difficult. For example, although the presence of the FCS promotes rapid cell entry and thus leads to increased infectivity, S‐proteins not pre‐activated by furin during viral assembly, or not processed by TMPRSS2, can still be cleaved at both S1/S2 and S2’ by endosomal cathepsins, as the virion undergoes late‐entry via an alternative endosomal pathway.^36^, ^37^, ^38^, ^39^

### 2.5 Neutralizing antibodies recognize the S‐protein

With COVID‐19 spreading around the globe, interest also grew exponentially into the characteristics of humoral and cellular immunity against SARS‐CoV‐2. Of particular relevance were potential therapies using convalescent plasma^40^ and monoclonal antibodies (mAbs),^41^ so it became essential to understand which classes of neutralizing antibodies are generated against the S‐protein and the structural details of their binding. Although more recent clinical trials and meta‐analyses showed no benefit for convalescent plasma on the overall survival of patients with moderate and severe COVID‐19,^42^ neutralizing mAbs remain promising for vulnerable individuals, with a number of such formulations being used in clinical settings (for reviews on mAb therapies in COVID‐19 see^43^, ^44^).

As mentioned previously, the heavy lifting (up to 90%) of viral neutralization in COVID‐19 is done by a minority of antibody species targeting the RBD,^18^, ^45^ the most potent of which contact the RBM specifically.^46^ These antibodies can be organized into four classes (I‐IV) based on their ability to recognize the ‘up’ or both the ‘up’ and ‘down’ positions of the RBD and to interfere with ACE2 binding^47^ (for reviews of antibody types see,^44^, ^48^). For example, five neutralizing mAbs from COVID‐19 patients bound to different regions of the RBD on each protomer but only two directly competed with ACE2, whereas the others merely sterically hindered receptor interaction (Figure 1).^49^ Similarly, an mAb (S309) originating from memory B cells of a SARS survivor bound to a different epitope than the receptor‐binding site and did not compete with ACE2 for S‐protein binding; however, it was capable of neutralization through indirect mechanisms, such as cross‐linking of S‐proteins or aggregation of viral particles.^50^ In fact, S309 has since proven highly efficient not just across SARS virus species, but also across SARS‐CoV‐2 variants and was further developed for treatment of high‐risk COVID‐19 under the name sotrovimab/VIR‐7831.^46^

Interestingly, deep mutational scanning and cryo‐EM revealed an almost inverse relationship between breadth and neutralization potency, with anti‐RBM antibodies being most neutralizing, but more vulnerable to escape mutations and with relatively low affinity across sarbecovirus species. Conversely, antibodies against conserved core regions of the RBD are less potent, but with higher cross‐reactivity and resistance to escape.^46^ Although likely rare, antibody species which offer the optimal balance between these properties have been shown to exist and represent likely candidates not only for therapies against COVID‐19, but also for other potential emerging coronavirus diseases.^46^, ^51^

The range of immune responses is made even more diverse by the presence of highly efficient anti‐NTD antibodies, which may represent up to 20% of neutralizing species.^52^ Despite the presence of six antigenic sites within the NTD (labelled *i*‐*vi*), all neutralizing antibodies screened against this region appear to contact supersite *i*, at the top of the NTD.^52^ It should be noted that the mechanisms through which neutralization occurs remain speculative.^53^ It was suggested that binding of mAbs to the NTD hinders required conformational changes in the trimer during infection by inhibiting proteolytic activation, preventing interactions with receptors other than ACE2, or simply blocking membrane fusion.^52^, ^54^ Not all anti‐NTD antibodies are beneficial, however. There is convincing evidence that a subcategory, which binds to a small region at the NTD surface, is infection‐enhancing. The proposed mechanism is that when divalent enhancing antibodies bridge adjacent S‐proteins, the NTD region is forced away from the RBD, which allows the RBD to switch to the open conformation, thereby increasing ACE2 binding.^55^

Overall, understanding how antibodies bind the S‐protein using cryo‐EM and other techniques helped shape a few key concepts and future research directions: (1) the RBD region of the S‐protein is immunodominant supporting the development of RBD‐based vaccines; (2) the high diversity of antibodies should encourage testing of antibody cocktails to minimize immune escape; (3) therapies should not only focus exclusively on antibody binding affinity, but also on breadth and their resistance to immune escape.

### 2.6 The S‐protein mutates under selective pressure

**FIGURE 3:**
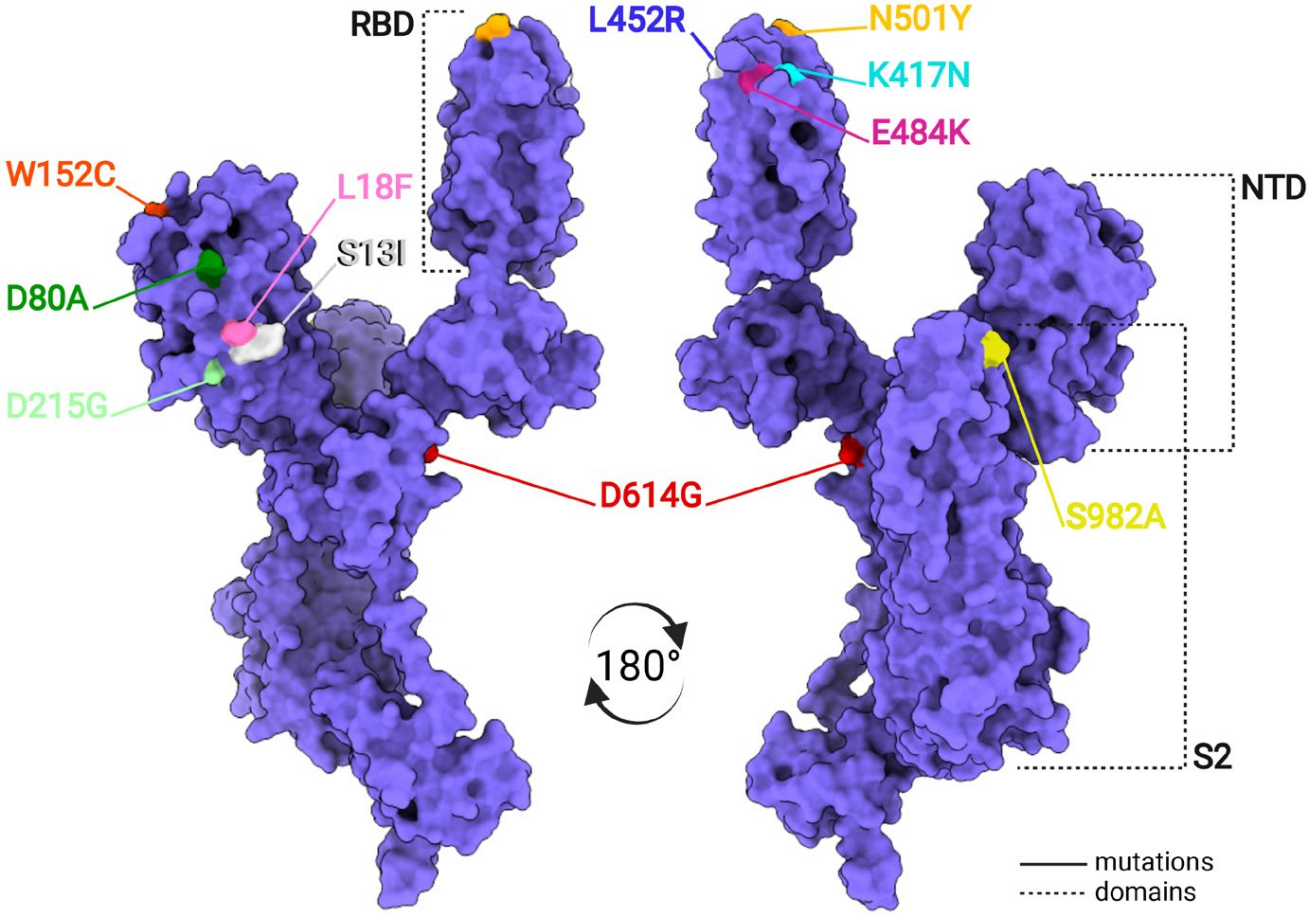
Locations of key mutations within the RBD and NTD domains of the S‐protein protomer. Diagrams are based on the cryo‐EM 3D structure deposited to PDB at 6VSB. RBD—*receptor*‐*binding domain*, NTD—*N*‐*terminal domain*. A—alanine; C—cysteine; D—aspartic acid; E—glutamic acid; G—glycine; F—phenylalanine; I—isoleucine; K—lysine; L—leucine; N—asparagine; R—arginine; S—serine; T—threonine; Y—tyrosine; W—tryptophan

Since the beginning of the pandemic of big concern were potential mutations within key S‐protein epitopes, which would affect antibody binding and MHC‐I^1^‐based cytotoxic lymphocyte surveillance. Through most of 2020 SARS‐CoV‐2 showed relatively high genomic stability, with the notable exception of an aspartic acid to glycine substitution within the S‐protein (D614G, Figure 3).^56^ This variant was first detected in late January 2020, but that has since become ubiquitous. Analysis of a 3.7 Å reconstruction of the D614G S‐protein revealed that the mutation loosens inter‐protomer interactions, promoting a higher proportion of open conformations (one, two or three ‘up‐RBDs’) as compared to the non‐mutated S‐protein (PDB 6XS6).^57^ This makes the S‐proteins more fusion‐prone and explains the increased infectivity of D614G variants.^57^

The mutational landscape abruptly changed towards the end of 2020. A global increase in selection pressure led to the emergence of more competitive virus variants, sharing the N501Y mutation of the RBM (an asparagine residue substituted to tyrosine, see Figure 3).^56^ High‐resolution cryo‐EM structures (2.9 Å, PDB 7MJM and 3.3 Å, PDB 7EDJ) of the mutated trimer complexed with ACE2 revealed that Y501 protrudes within a cavity of the receptor, allowing an additional interaction with Y41 of ACE2.^58^, ^59^ Although this suggested that N501Y confers increased stability and binding efficiency, it only appeared to alter the neutralizing capability of an antibody that specifically covers the epitope containing N501Y (Figure 1).^58^, ^59^

Additional amino acid deletions and substitutions continued to accrue, predominantly not only affecting the S‐protein and influencing not just ACE2 binding affinity, but also facilitating immune escape.^56^, ^60^ First to become widespread were variants Alpha (B.1.1.7 or 501Y.V1) and Beta (B.1.351 or 501Y.V2), and concerns were raised immediately to their infectivity and ability to avoid natural or vaccine‐induced immunity. Indeed, cryo‐EM rapidly revealed key differences to the ancestral S‐protein, which could account for observed changes in COVID‐19 pathophysiology. On one hand, the increased infectivity and broader cell tropism of the Alpha variant were explained by a higher predisposition of the S‐protein for an open architecture; mainly, the result of mutation S982A (Figure 3) which reduces constraints on the RBDs to remain in the ‘down’ conformation.^61^, ^62^ On the other hand, Beta presented a reconfiguration of NTD loops due to a triple residue deletion and three point mutations (L18F, D80A and D215G, see Figure 3) and this could significantly diminish the neutralizing activity of antibodies targeting that region.^61^ In the RBD, aside from the staple N501Y mutation, the Beta and Gamma (P.1) variants also contained the infamous K417N/K417T and E484K mutations (Figure 3). The latter appears to destabilize the RBD tip, with two consequences: (1) a reduction in RBD‐RBD interactions allowing an increase in ‘up’ RBDs and (2) a reduction in binding of important anti‐RBD antibodies due to loss of native conformation.^61^, ^62^ In neutralization assays, this translated up to a remarkable 115‐fold reduction in neutralization by plasma from some convalescent individuals.^18^

Despite downgraded from variant of concern to variant of interest by the CDC^63^ due to reduction in prevalence, the Epsilon variant (B.1.427/B.1.429) has nevertheless also been scrutinized for its ability to avoid immune surveillance. Epsilon harbours the L452R mutation in the RBD and the S13I and W152C mutations in the NTD (Figure 3). Whereas L452R sterically impedes binding of at least some anti‐RBD antibodies, S13I and W152C allow escape from all neutralizing antibodies tested against supersite *i* of the NTD. Indeed, an antibody‐stabilized cryo‐EM structure of the Epsilon S‐protein revealed that these mutations induce severe disordering of the N‐terminal region (Figure 1, PDB 7N8H, 7N8I).^64^

During the summer of 2021, evidence mounted supporting higher transmissibility for the Delta (alternatively B.1.617.2) variant and its ability to significantly elude natural or vaccine‐induced immunity.^63^ However, Delta does not hide its tricks within the RBD, which is relatively conserved. Instead, it displays a modified NTD, in which mutations T19R, G142D and E156G and deletions F157 and R158 lead to a conformational shift of a β‐strand comprising key epitopes (Y144, K147, K150 and W152) and a reconfiguration of the 173–187 loop, both critical regions for anti‐NTD recognition (PDB 7SBK, 7SBL and 7SBO).^65^, ^66^ Worryingly, in their pre‐print, Liu et al. suggest that epitopes targeted by infectivity‐enhancing anti‐NTD antibodies are conserved in Delta and may thus offset anti‐RBD activity, especially when anti‐RBD antibody titers fall.^65^

Some data are encouraging, however. Firstly, at least some anti‐RBD antibodies from individuals recovered from COVID‐19 retain the ability to neutralize across emerging variants, by exhibiting reduced contact to mutation‐susceptible residues, such as E484 or L452.^46^, ^51^, ^67^, ^68^ An antibody shown by cryo‐EM to effectively ‘dodge’ these residues has also been generated in mice.^69^ Secondly, although mRNA vaccines appear to preferentially elicit anti‐RBD neutralizing species, these antibodies are highly diverse, recognize both the ‘up’ and ‘down’ positions of the RBD and have broader specificity to that region than antibodies induced by natural infection, thus making them less vulnerable to mutations.^45^, ^70^ Until new vaccines or boosters are made available, we should rest on the idea that some immunity is likely better than no immunity.

At the time of writing, it remains unclear how newer variants of interest (Mu^71^ and C.1.2^72^) influence S‐protein architecture and its interactions.

### 2.7 The N, M and E proteins are more elusive

Despite the success in characterizing the S‐protein, approaching the other three structural proteins by SPA or cryo‐ET is significantly more difficult. For example, studies of the M‐protein are hindered by the fact that it does not assemble into a visible matrix layer as in other enveloped viruses, like influenza.^32^ On the other hand, the E‐protein is too small (just above 8 kDa), whereas the N‐protein is intrinsically disordered.^73^ Until now, these proteins have mostly been approached using other structural biology methods, for both SARS‐CoV‐2 and related coronaviruses (for a comprehensive review, see^74^). Coupling these proteins to ligands or fusing them to molecular scaffolds^75^ may expand the reach of cryo‐EM studies. For example, Chai et al. recently investigated the molecular complex formed between a SARS‐CoV‐2 E‐protein C‐terminal peptide and a fragment of the PALS1^2^ cell junction protein. The study offered a structural basis for how the E‐protein relocates PALS1 and thus disrupts the polarity complex and tissue structure during infection (PDB 7M4R).^76^

Cryo‐ET has also proven useful in revealing the internal organization of the virus, specifically the geometrical arrangement of the ribonucleoproteins (RNPs), which are formed by the association of N‐protein oligomers and viral RNA (vRNA) molecules. There are, on average, 30–38 RNPs within each virion, 14–15 nm across, organized as tightly packed stacks and likely following a beads‐on‐a‐string architecture, with N‐proteins linking adjacent RNPs.^32^, ^35^

## 3 NONSTRUCTURAL PROTEINS AS TARGETS FOR DRUG DEVELOPMENT

Once SARS‐CoV‐2 has infected a cell, a cascade of intracellular events begins, comprising replication of vRNA and synthesis of structural proteins which will further assemble into new virions. This complex process is made possible by a set of 16 non‐structural proteins (NSPs), encoded by two open reading frames (ORF1a and ORF1b) of the SARS‐CoV‐2 genome. The initial translation products of ORF1a and ORF1b are two large polyproteins (designated pp1a and pp1ab), which are cleaved by host and viral proteases to release the individual NSPs required for replication. These NSPs have a miriad of functions, some poorly understood, ranging from proteolysis of viral polyproteins to the formation of a replisome‐like multi‐protein complex that performs replication and transcription of vRNA (for detailed reviews on the roles of coronavirus NSPs see,^77^, ^78^).

RNA replication is thought to occur in large (~300 nm), specialized intracellular compartments called double‐membrane vesicles (DMVs). These arise through extensive rearrangements of endoplasmic reticulum (ER) cisternae, a process made possible by the interaction of nsp3, nsp4 and nsp6 embedded within ER membranes.^79^ In situ cryo‐ET has helped reveal morphological details of these DMVs, at high resolution. Within each DMV are numerous vRNA strands, specifically A‐form double stranded RNA, while single‐stranded RNA and densities suggestive of replication‐transcription complexes (RTCs) appear absent.^32^ This may be explained by the specific localization of RTCs at the inner membrane of DMVs,^32^ where they allow rapid escape of synthesized vRNA to the cytosol via molecular pores. Indeed, these pores, likely assembled from nsp3 hexamers, have been shown to interact with conspicuous densities on the DMV lumenal side, potentially RTCs.^80^ Exported vRNA then joins cytosolic N‐proteins to form RNPs and these further associate with envelope proteins in the ERGIC. Intracellular virions are generated through budding in ERGIC lumens.^32^, ^80^

The critical role that the RTC plays in infection makes it an attractive target for antiviral therapies, and numerous cryo‐EM studies have investigated the structure and function of the proteins that make up the SARS‐CoV‐2 replication machinery (mainly nsp7, nsp8, nsp9, nsp12 and nsp13).^81^, ^82^, ^83^, ^84^, ^85^, ^86^, ^87^, ^88^ Of these, nsp12 was unsurprisingly of the highest priority. Also known as the RNA‐dependent RNA polymerase (RdRp), it resides at the core of the RTC, driving vRNA synthesis. Early in 2020, the first structure of the SARS‐CoV‐2 RdRp with cofactors nsp7 and 8 was resolved at 2.9 Å resolution, revealing it adopts an almost identical architecture to that of the SARS‐CoV‐1 RdRp (Figure 1, PDB 6M71, 7BTF).^81^ Briefly, nsp12 comprises an N‐terminal extension domain (nidovirus RdRp‐associated nucleotidyltransferase or NiRAN) and the C‐terminal RdRp domain, which is organized three‐dimensionally as a closed right hand, with the finger‐palm‐thumb subdomains enclosing and stabilizing the vRNA strand.^81^

Critically, the polymerase active site, located within the palm subdomain of RdRp, proved highly similar to those of HCV or poliovirus, suggesting vulnerability to known modified nucleotide analogues, such as Remdesivir.^81^ In their triphosphate form, these drugs enter the active site of RdRp, where they compete with cellular nucleotide triphosphates (NTPs) to incorporate into the nascent vRNA chain, thus blocking synthesis.^89^ Indeed, not long after, another study clarified that a single Remdesivir monophosphate is covalently integrated into the vRNA strand at the first replicated base pair, thus prematurely terminating synthesis (PDB 7BV2).^82^ Although in vitro data offered reasons for optimism and despite FDA approval in late 2020,^90^ Remdesivir failed to live up to expectations in clinical studies. At the time of writing, a meta‐analysis of 7452 patients suggested it has little or no effect on mortality in COVID‐19.^91^

Favipiravir is another widely used nucleotide analogue investigated for its ability to inhibit SARS‐CoV‐2 polymerase activity. Although essentially asking the same question, two studies looking at the structural basis of inhibition by this drug arrived at different results. One found that the triphosphate form of Favipiravir is mostly in a catalytically nonproductive conformation within the RdRp active site, which leads to inefficient incorporation into the vRNA strand (PDB 7AAP).^92^ By contrast, another study found that Favipiravir is in fact recognized within the active site and incorporated into nascent vRNA (PDB 7CTT).^93^ A likely explanation is that the latter study captured the minority of productive conformations of the triphosphate within RdRp.^92^

Lastly, a surprising drug candidate to be screened against the SARS‐CoV‐2 RdRp was the relatively obscure suramin, a urea‐based compound traditionally used for treating trypanosomiasis, but more recently also evaluated for some viral diseases due to its ability to inhibit viral entry and release (^94^ and references cited therein). A 2.57 Å structure revealed that two suramin molecules fit within the RdRp, where they form multiple bonds with surrounding residues, including at the catalytic site (PDB 7D4F). This arrangement directly hinders interactions between vRNA and the active site, leading to inhibition of polymerase function.^94^ Suramin has yet to be evaluated for COVID‐19 through clinical trials.

Although the SARS‐CoV‐2 polymerase is the most obvious target for antiviral therapies, the structures of other NSPs have also been resolved by cryo‐EM, to facilitate potential drug design. One is nsp1, a protein that is now shown to completely obstruct the mRNA entry channel of ribosomes, inducing preferential suppression of host‐cell protein translation and implicitly, of host antiviral responses (PDB 6ZLW, 6ZOJ).^95^, ^96^ Another is nsp15, a hexamer that cleaves the polyuridines which accumulate at the 5’‐end of vRNA intermediates as these can activate host responses (PDB 7K0R).^97^

## 4 FINAL THOUGHTS

It is inevitable that SARS‐CoV‐2 will continue to adapt and new variants are likely to emerge in the near future. At least until artificial intelligence structural predictions mature and are able to accurately suggest outcomes for complex protein interactions, cryo‐EM SPA will remain at the forefront of molecular virology. It will continue to offer a glimpse into how novel mutations change the behaviour of SARS‐CoV‐2, its affinity to ACE2 and potentially other cell receptors, its interaction with antibodies and its ability to replicate inside cells. Cryo‐ET can also be expected to further shed light on changes in cell morphology during the viral infectious cycle, which still remain relatively unexplored. Last but not least, cryo‐EM has set an important precedent in COVID‐19, as it significantly closed the gap between bench and bedside: It directly supported and will continue to support the development of much needed antiviral therapies and vaccines.

## CONFLICT OF INTEREST

The authors declare no conflicts of interest.

## AUTHOR CONTRIBUTION

**Tudor Emanuel Fertig:** Conceptualization (lead); Data curation (lead); Funding acquisition (supporting); Investigation (lead); Methodology (lead); Project administration (lead); Resources (lead); Supervision (lead); Validation (lead); Visualization (lead); Writing – original draft (lead); Writing – review & editing (lead). **Leona Chitoiu:** Conceptualization (supporting); Data curation (equal); Investigation (supporting); Methodology (supporting); Project administration (supporting); Resources (supporting); Validation (supporting); Visualization (supporting); Writing – original draft (supporting); Writing – review & editing (supporting). **George Terinte‐Balcan:** Conceptualization (supporting); Data curation (supporting); Investigation (supporting); Methodology (supporting); Project administration (supporting); Resources (supporting); Validation (supporting); Visualization (supporting); Writing – original draft (supporting); Writing – review & editing (supporting). **Victor Eduard Peteu:** Conceptualization (supporting); Data curation (lead); Investigation (supporting); Methodology (supporting); Project administration (supporting); Resources (supporting); Validation (supporting); Visualization (supporting); Writing – original draft (supporting); Writing – review & editing (supporting). **Daciana Marta:** Conceptualization (supporting); Data curation (supporting); Funding acquisition (supporting); Investigation (supporting); Methodology (supporting); Project administration (supporting); Resources (supporting); Validation (supporting); Visualization (supporting); Writing – original draft (supporting); Writing – review & editing (supporting). **Mihaela Gherghiceanu:** Conceptualization (equal); Data curation (equal); Funding acquisition (lead); Investigation (equal); Methodology (equal); Project administration (equal); Resources (equal); Supervision (equal); Validation (equal); Visualization (equal); Writing – original draft (equal); Writing – review & editing (equal).

## Data Availability

Data sharing is not applicable to this article as no datasets were generated or analysed during the current study.

## ENDNOTES

^1^
MHC‐I ‐ class I major histocompatibility complex

^2^
PALS1 – protein associated with LIN7 1
